# Supplementary material for: Seroprevalence of COVID-19 infection in a rural district of South India: A population-based seroepidemiological study
Source: PLoS One. 2021 Mar 31;16(3):e0249247. doi: 10.1371/journal.pone.0249247 (PMC8011723; doi:10.1371/journal.pone.0249247)
Supplement: Attachment — Submitted filename: Response to reviewers.docx [file pone.0249247.s004.docx]

**Response to the comments of the editor and reviewer**

We thank the editor and reviewer for taking the time and going through our manuscript (PONE-D-20-39600). We appreciate the constructive feedback received to improve the quality of the paper. We have addressed the points raised and submitted a revised version of the manuscript with highlights. We have given below our response to the comments

**Editorial comments**

1. **Please ensure that your manuscript meets PLOS ONE's style requirements, including those for file naming.**

We have formatted the article according to the journal requirement and made changes in the file names

2. **Your ethics statement should only appear in the Methods section of your manuscript. If your ethics statement is written in any section besides the Methods, please move it to the Methods section and delete it from any other section.**

We have deleted the ethics statement from other section and kept it only in the methods section

3. **You may seek permission from the original copyright holder of Figure 1 to publish the content specifically under the CC BY 4.0 license.**

We have obtained permission from the original copyright holder for Figure 1 and submitted

**Reviewer's comments**

1. **The manuscript is well written except for minor corrections and clarifications**

We are grateful for this feedback and appreciate the efforts in reviewing our manuscript

2. **The results of association between different variables and seropositivity can be presented as a table**

Thank you for this comment. We have now presented this analysis as a new Table.3

3. **What is the justification for selecting three months for symptom recall?**

We understand that we have used 3 months recall period even though a 15 days recall period is generally recommended for eliciting morbidity. We assumed that people would recall their symptoms related to COVID for a longer period due to the unusually significant nature of this pandemic and the attention it had received from media. However, this could have resulted in recall bias. We have mentioned this as one of the limitations (347-50, page 16). At the same time, we did not want to go beyond three months as people tend to forget minor symptoms.

4. **There are minor typos throughout the manuscript that need to be fixed**

We have gone through the manuscript carefully fixed all the typos.
